# Ventilation Imaging of the Lung at 0.55T With Continuous Slice Cycling

**DOI:** 10.1002/mrm.70436

**Published:** 2026-05-14

**Authors:** Andrea Leuthard, Grzegorz Bauman, Katrin E. Hostettler, Maurice Pradella, Oliver Bieri

**Affiliations:** ^1^ Department of Biomedical Engineering University of Basel Allschwil Switzerland; ^2^ Department of Radiology, Division of Radiological Physics University Hospital Basel Basel Switzerland; ^3^ Clinics of Respiratory Medicine University Hospital Basel Basel Switzerland; ^4^ Department of Radiology University Hospital Basel Basel Switzerland

**Keywords:** low‐field, lung, MRI, non‐contrast‐enhanced, ventilation

## Abstract

**Purpose:**

To propose and evaluate a novel method for pulmonary ventilation imaging, offering considerably improved SNR.

**Methods:**

A continuous slice cycling (CSC) acquisition scheme is proposed to exclusively capture signal modulations from respiratory motion with increased SNR. To this end, a set of single‐shot images is acquired at multiple interleaved slice positions. This scheme offers substantially increased image‐to‐image time‐intervals at every slice position, thus allowing near full longitudinal recovery between repeated acquisitions but without losing scan efficiency. Ventilation images are generated from the registered CSC image time series using the coefficient of variation and compared to conventional functional lung MRI using a time‐resolved series of 2D images. Functional lung MRI was performed in 7 volunteers and 11 patients at 0.55T using balanced steady state free precession (bSSFP). Six coronal slices with 100% slice spacing were acquired with an image‐to‐image interval of 2 s, yielding a total scan time of 3:20 min for 100 cycles.

**Results:**

For the proposed 2 s time‐interval at 0.55T, almost full signal recovery can be achieved. As a result, CSC centric imaging yields a more than two‐fold SNR increase as compared with contemporary functional lung imaging methods. Excellent agreement was observed between ventilation images from CSC and those from standard functional lung MRI.

**Conclusion:**

CSC holds strong potential for ventilation MRI with improved sensitivity, particularly in diseases marked by reduced local tissue density.

## 1 Introduction

Functional imaging of the lung can be performed by various MRI methods. These include techniques for direct ventilation mapping, such as hyperpolarized 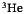 [1, 2, 3] or 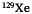 [4, 5], or fluorinated gas MRI [6, 7]. Alternatively, native—that is non‐contrast‐enhanced—free‐breathing 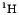 imaging methods can assess lung function indirectly from a time‐resolved series of snapshot‐like 2D images, as proposed by Zapke et al. two decades ago [8]. However, spatial coherence must be achieved using a suitable image registration method to compensate for the respiratory motion.

Over the years, several post‐processing methods have been proposed to retrieve 2D lung functional information from the observed signal modulations in the lung tissue. These include a signal analysis in the Fourier domain (using Fourier Decomposition; FD) [9], in the time domain (using Matrix Pencil analysis; MP [10] or dynamic mode decomposition [11]), using retrospective sorting (Phase‐REsolved FUnctional Lung MRI; PREFUL) [12], or self‐navigation (SElf‐gated Non‐Contrast‐Enhanced FUnctional Lung MRI; SENCEFUL) [13]. For the acquisition of the time‐resolved scan, generally, two pulse sequence kernels are in use: either RF spoiled gradient echo (GRE) [14] or balanced steady‐state free precession (bSSFP) [15]. PREFUL and SENCEFUL are typically based on GRE, whereas FD MRI and MP MRI are based on bSSFP [9, 10, 12, 13].

Compared with gaseous tracer methods, native functional lung MRI relies on standard clinical MRI hardware and does not require complex experimental setups. This makes native functional lung MRI highly versatile, easily accessible and more cost‐effective. A major disadvantage of all proton‐based lung imaging methods, however, is that they need to handle the most fundamental challenge associated with the lung [16]: physiological motion and low signal due to low proton density in combination with a myriad of air‐tissue interfaces on the mesoscopic length scale causing a rapid $T_{2}^{*}$‐related signal decay.

In this context, the use of bSSFP for functional lung MRI is particularly noteworthy, since it offers the highest signal per unit time of all imaging sequences [17]—a key to push the SNR limits in lung imaging. Unfortunately, bSSFP is sensitive to field inhomogeneities which can lead to the appearance of so‐called banding artifacts in the image at off‐resonance frequencies of ±n/(2TR), where n is an odd integer [17]. For short enough TR, however, not only banding effects can be successfully mitigated but also a spin‐echo like gradient echo is formed [18, 19]. Thus, bSSFP does not suffer from the pronounced rapid $T_{2}^{*}$‐related signal decay. Robust functional lung MRI with bSSFP, however, has only become available due to the development of ultra‐fast bSSFP, providing a TR close to 1 ms and thus offering artifact‐free bSSFP at 1.5T [20]. This constraint becomes relaxed with decreasing field strength, and low field MRI has shown excellent prospects for lung MRI with bSSFP [21, 22]—but only at the expense of generally lower SNR. Nevertheless, TR and thus the readout must be kept short.

At high‐magnetic fields, such as 3T and above, however, bSSFP currently fails to provide a short enough TR to achieve artifact‐free imaging of the lung due to increased field inhomogeneities [23]. Thus, functional lung imaging must be performed with GRE but from its rapid signal decay, both the readout and TE must be kept short. This overall results in a low SNR for 2D imaging, since the lower bound of TE is generally limited by the slice selection. Alternatively, and independently of the field strength and sequence used, averaging might be used to increase the SNR. This will, however, inevitably prolong the acquisition time which is typically incompatible with breath‐holding maneuvers as well as for functional lung MRI that relies on a rapid sampling of the respiratory and perfusion‐related signal changes.

Overall, native functional lung MRI has shown excellent prospects for broad clinical application in a wide range of pulmonary diseases, such as cystic fibrosis [24, 25, 26], primary ciliary dyskinesia [27], chronic obstructive pulmonary disease [28, 29], asthma [30], COVID‐19 [31], or lung cancer [32]. Despite all the technical advances in hardware, sequence design and image post‐processing, functional lung MRI—like all lung MRI methods—remains challenged by the intrinsically low proton density of the lung parenchyma. This becomes especially critical for assessing so‐called “minus‐pathologies” [16], which are characterized by a decrease in local tissue density, for example in emphysematous tissue destruction or by hyperinflation caused by air‐trapping [16].

In this work, we present a novel 2D acquisition strategy to considerably boost the SNR from the lung tissue for ventilation MRI. Unlike conventional methods that acquire time‐resolved data at one single slice location in quasi steady‐state, the proposed technique employs a continuous slice cycling (CSC) scheme across multiple slice locations. By cycling through multiple slices, close to full recovery of magnetization can be achieved prior to any image acquisition, leading to enhanced signal and thus improved SNR. The prospects of CSC‐based ventilation MRI of the lung are demonstrated in healthy volunteers and in a small cohort of patients using bSSFP at 0.55T.

## 2 Methods

### 2.1 CSC

**FIGURE 1:**
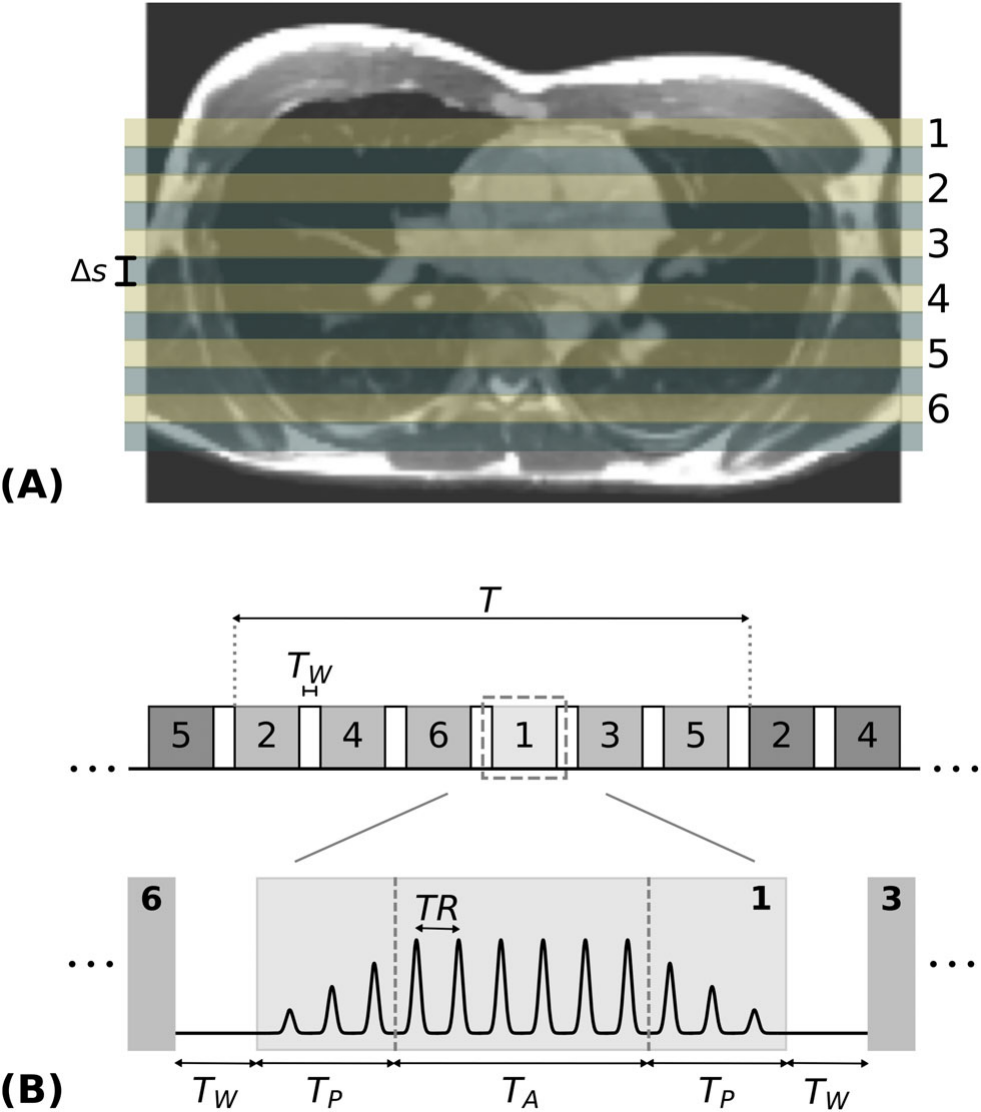
Illustration of the proposed CSC methodology. (A) A total of n=6 equidistant coronal slices are acquired using 100% slice spacing (Δs) to mitigate possible signal interference from adjacent slices. (B) The slices are acquired in an interleaved manner, as indicated by the slice numbering. At each slice position, a snapshot‐like image acquisition is performed using a SSFP sequence (only RF pulses are shown) with a repetition time of the SSFP kernel of *TR*. The duration of one cycle through the n slice positions is indicated by the time T, which is equivalent to the sampling period of the breathing motion at one specific slice location. The time required for sampling one image is indicated by $T_{A}$. For balanced SSFP, typically, some preparation periods ($T_{P}$, preceding and following the image encoding) are used, such as by using a linear ramp up and ramp down of the flip angle, as indicated. Moreover, an optional waiting time $T_{W}$ allows to adjust the sampling period.

A CSC scheme is proposed (see Figure 1) to capture respiratory motion‐related signal modulations of the lung tissue over time with increased sensitivity. To this end, the CSC sequence is designed to allow for each slice position nearly full magnetization recovery between snapshot‐like image acquisitions. As a consequence, respiratory motion‐related changes in the local proton density (reflecting ventilation) are expected to be detected with maximum signal, while sensitivity to blood perfusion should, by design, be minimal.

Overall, CSC has some similarity to an interleaved multi‐slice acquisition and is shown in Figure 1A. In contrast to conventional multi‐slice acquisitions, however, a set of single‐shot images is acquired at n different positions. The acquisition is repeated N times, overall resulting in a total of n×N images. To mitigate any possible signal interference, gaps between adjacent slices and an interleaved slice ordering are used.

The timing of the CSC acquisition is shown in Figure 1B. Each image acquisition contains: (i) an optional preparation period $T_{P}$ before and after the image encoding, and (ii) the actual image sampling with duration $T_{A}$. The image acquisition is followed by an optional waiting time $T_{W}$. At each slice position, the signal variation of the lung tissue is thus sampled N times with a time period $T=n\cdot \left(T_{A}+2\cdot T_{P}+T_{W}\right)$. For the image acquisition, a SSFP sequence is used, as in related works [9, 10].

### 2.2 Imaging Protocols

MRI scans were performed on a commercially available 0.55T low‐field MR system (MAGNETOM Free.Max, Siemens Healthineers, Forchheim, Germany) equipped with low‐performance gradients (25 mT/m amplitude, 40 mT/m/s slew rate). The integrated body coil was used for RF transmission and a combination of a 6‐channel chest array coil and a 6‐channel spine coil was used for signal reception.

For CSC, a total of n = 6 coronal slices with 10 mm thickness were acquired using 100% slice spacing, covering 110 mm (see Figure 1A). Each image was encoded using a bSSFP kernel with a TR of 2.9 ms for a field‐of‐view of $475\times 475\;{\mathrm{mm}}^{2}$ with 128×128 matrix size (interpolated to 256×256 and 1.86 mm isotropic resolution). For excitation, a sinc‐shaped RF pulse with a time‐bandwidth product of 2, a flip angle of 60°, and a pulse duration of 0.6 ms was used. The readout bandwidth was 1002 Hz/pixel. One image was encoded in $T_{A}=185.6\;\mathrm{ms}$ using parallel imaging with GRAPPA factor 2. A separate 2D GRE reference scan preceded the CSC acquisition for estimation of the coil sensitivities (24 central lines per slice; 69.6 ms duration per slice). For preparation, a linear ramp up of 10 flip angles was used to mitigate initial transient signal oscillations and a linear ramp down of 10 flip angles to refocus the magnetization along the longitudinal axis. Image encoding was performed either with a linear or a paired centric view ordering [33].

The sampling period T was fixed to 2 s, resulting in a $T_{W}$ of 90 ms. A total of N=100 cycles were acquired, yielding an acquisition time of 3:20 min for 600 images.

In addition, for selected CSC slice positions, time‐resolved 2D imaging was performed using the same bSSFP acquisition (including magnetization preparations) and image reconstruction, ensuring equivalent image noise. This allows an estimation of the relative increase in the SNR (rSNR) for the CSC scans as compared to the time‐resolved scans based on their relative signal strength. For subsequent MP analysis, however, 140 images were acquired with a linear view ordering and a waiting time of 90 ms (resulting 3 frames/s; as usual [9]). Scanning took 47 s per slice position.

### 2.3 Volunteer and Patient Scans

Seven volunteers (27.3 years ± 2.8 years) were scanned with the proposed CSC acquisition protocol and multiple time‐resolved 2D scans (at matched slices positions). In one volunteer, scanning was additionally performed in breath‐holding, using (i) one CSC scan with N = 20 cycles in 41 s, (ii) multiple time‐resolved 2D scans in 33 s with a waiting time of 90 ms, and (iii) multiple time‐resolved 2D scans using N = 10 cycles and variable waiting times $T_{W}$ = 0, …,3 s. For all volunteer scans, the slice thickness was fixed to 10 mm.

In addition, the CSC acquisition and a series of time‐resolved 2D scans were added to an exploratory clinical lung MRI protocol in 11 patients with known pulmonary diseases (pulmonary fibrosis [75 years, f]; lung cancer [66 years, f]; T2‐low asthma [52 years, f]; actinomycosis infection [61 years, m]; scleroderma‐associated interstitial lung disease [64 years, f]; connective tissue disease‐associated interstitial lung disease [79 years, f]; combined pulmonary fibrosis and emphysema [64 years, m]; idiopathic pulmonary fibrosis [67 years, f]; suspicion of early interstitial lung disease [78 years, m]; suspicion of neuromuscular disease [37 years, f]; Langerhans cell histiocytosis [76 years, m]). For the CSC scan, the slice thickness was fixed at 10 mm (four patients), whereas the slice thickness for the time‐resolved 2D scan was initially set to 10 mm but was subsequently increased to 12 mm.

The local Ethics Committee approved the study and written informed consent was obtained from all volunteers and patients.

### 2.4 Simulations

CSC with bSSFP was simulated for lung tissue at 0.55T under ideal conditions (on‐resonance, instantaneous RF pulses, and neglecting motion and diffusion) using 3×3 rotation and relaxation matrices from the piece‐wise constant integrated Bloch equation [34]. The T2/T1 was set to 61 ms/971 ms [21]. Exactly the same settings as specified for the CSC protocol were used. Simulations were performed for a single slice but with variable sampling periods T. The flip angle profile was taken into account using the small tip angle approximation [35].

### 2.5 Ventilation Imaging With CSC

For the analysis of the signal, spatial coherence must be achieved for the images acquired at different breathing phases. Thus, the acquired images were first co‐registered, for each slice position separately, to one fixed image at intermediate lung position [12, 36]—using a regularization method that is based on adaptive anisotropic graph diffusion [37]. The intermediate respiratory state was selected deterministically by extracting a surrogate respiratory signal from the time‐series (mean pixel intensity per frame) and consistently identifying the specific frame closest to the global temporal average of this surrogate signal. Furthermore, a mask of the lung tissue was generated for all slice positions from the corresponding image in the intermediate lung position.

Generally, the proposed CSC acquisition scheme aims for a full recovery of the magnetization in‐between image acquisitions performed at identical slice positions. For a voxel, the set of signals $S_{j}$ (acquired at times $t_{j}$) should thus only become modulated by the periodic inflation and deflation of the lung tissue, taking the form

(1) $$S_{j}=\left\langle S\right\rangle +V_{j}+N_{j}$$

where $N_{j}$ represents a noise term, ⟨S⟩ is the mean signal over time and $V_{j}$ refers to the actual signal variation, as introduced by the respiratory cycle.

We now rewrite Equation (1) to take the form:

(2) $$\frac{S_{j}-\left\langle S\right\rangle }{\left\langle S\right\rangle }=v_{j}+n_{j}$$

using the definitions

(3) $$v_{j}≔V_{j}/\left\langle S\right\rangle \;n_{j}≔N_{j}/\left\langle S\right\rangle$$

where $v_{j}$ is the actual fraction ventilation. Note that this is identical to the definition used by Zapke et al. [8], if we replace ⟨S⟩ with the signal at expiratory state $S_{\exp}$.

Since normal breathing rates are reported to range between 12 and 20 times per minute [38] (with corresponding sampling frequencies $f_{B}$ ranging from 0.2 s^−1^ to about 0.33 s^−1^), the image sampling rate at a fixed slice position $f_{s}≔T^{-1}$, will most likely not fulfill the Nyquist criterion; requiring breathing rates $f_{b}<0.5f_{s}$. This will, in general, prevent a simple analysis of the signal time course to retrieve fractional ventilation, such as used with MP MRI, or a sorting of the images to a harmonic model function with arbitrary chosen respiration frequency to retrieve their breathing phase, as performed with PREFUL [12].

Thus, discarding the time information, we calculate the variance of Equation (2), yielding

(4) $$\mathrm{Var}\left(\frac{S_{j}-\left\langle S\right\rangle }{\left\langle S\right\rangle }\right)=\left\langle v^{2}\right\rangle +\left\langle n^{2}\right\rangle$$

where we have assumed that ⟨v⋅n⟩≈0. Realizing that the left‐hand side of Equation (4) is the square of the coefficient of variation, CV, for the signals $S_{j}$, we thus get

(5) $${\mathrm{CV}}^{2}=\left\langle v^{2}\right\rangle +\left\langle n^{2}\right\rangle$$

where we have assumed a similar noise level for all voxel positions ($\left\langle {\left(n_{x}\right)}^{2}\rangle \approx \langle n^{2}\right\rangle$).

In the following, we consider the signals $S_{1,j}$ and $S_{2,j}$ from two voxels with fractional ventilation $v_{1,j}$ and $v_{2,j}$, respectively. Specifically, we consider the ratio

(6) $$v_{1,j}/v_{2,j}=k$$

which should be a constant (k), since by design,

(7) $$\left\langle v_{1}\right\rangle =\left\langle v_{2}\right\rangle =\left\langle n_{1}\right\rangle =\left\langle n_{2}\right\rangle =0$$

and the breathing motion can locally only lead to a scaling in the fractional ventilation amplitude but should not affect its actual phase.

As a result,

(8) $${\mathrm{CV}}_{1}^{2}-\left\langle n^{2}\right\rangle =\left\langle v_{1}^{2}\right\rangle =k^{2}\left\langle v_{2}^{2}\right\rangle =k^{2}\cdot {\mathrm{CV}}_{2}^{2}-\left\langle n^{2}\right\rangle$$

and thus

(9) $$\frac{{\mathrm{CV}}_{1}}{{\mathrm{CV}}_{2}}=k=\frac{v_{1,j}}{v_{2,j}}$$

In summary, the coefficient of variation will not yield the fractional ventilation amplitude itself, but it is directly proportional to it.

Estimation of fractional ventilation amplitudes from the time‐resolved 2D image series was performed with MP decomposition [10] using an automated software pipeline—Truelung [36].

The ventilation maps obtained from the CV analysis were compared with those derived from the MP analysis of the time‐resolved acquisition by quantifying ventilation defect percentages. Voxels are classified as defects if their ventilation is below 70% of the median value of the respective map [24].

## 3 Results

**FIGURE 2:**
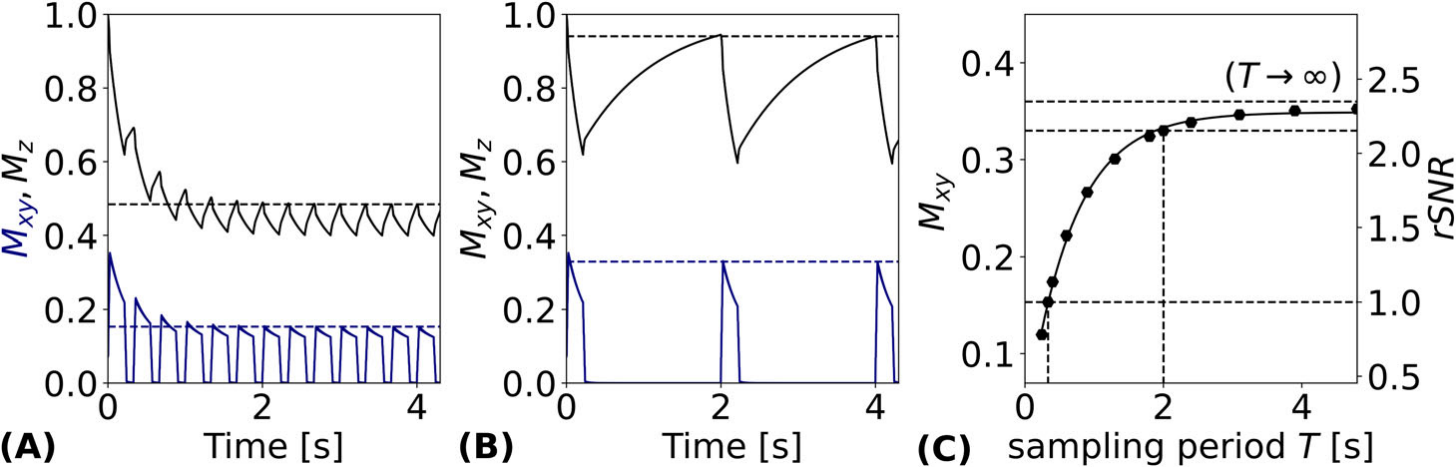
Simulation of the effect of the sampling period T on the CSC signal. (A, B) The transverse (magnitude $M_{\mathrm{xy}}$, blue) and longitudinal ($M_{z}$, black) magnetization are shown as a function of time for selected sampling periods of T = 0.33 s and T = 2 s, respectively. (C) Overall dependency of $M_{\mathrm{xy}}$ on T (relative to its value at T→∞) and of the predicted relative increase in the SNR (rSNR) for CSC on T, as compared to the time‐resolved scan.

The results of the simulation are summarized in Figure 2. For waiting times $T_{W}$ of 90 ms and 1758 ms with corresponding sampling times of 0.33 s and 2 s, the time‐course of the transverse ($M_{\mathrm{xy}}$) and longitudinal magnetization ($M_{z}$) are shown in Figure 2A,B. As expected, imaging is performed near steady state for short waiting times ($T_{W}\ll$ T1, Figure 2A), but reaches near equilibrium conditions for long sampling periods (Figure 2B). The dependency of $M_{\mathrm{xy}}$ with T is shown in Figure 2C (for a centric acquisition), together with the predicted rSNR increase. A non‐linear least squares fit indicates that the signal recovery closely follows a single exponential with an apparent rate of 1/0.66 s^−1^. As targeted, CSC imaging with a sampling period of T = 2 s thus achieves close to full recovery (about 94% of equilibrium magnetization $M_{0}$ for the simulated T1 and T2 values). Overall, the simulation predicts a rSNR increase for the proposed CSC acquisition (using T = 2 s), as compared to a time‐resolved scan ($T_{W}$ = 90 ms, T = 1/3 s) by a factor of 2.15 for centric acquisitions. The results from an in vivo scan in a healthy volunteer using a time‐resolved 2D scan but with linear view ordering and variable waiting times is shown in the Supporting Information.

**FIGURE 3:**
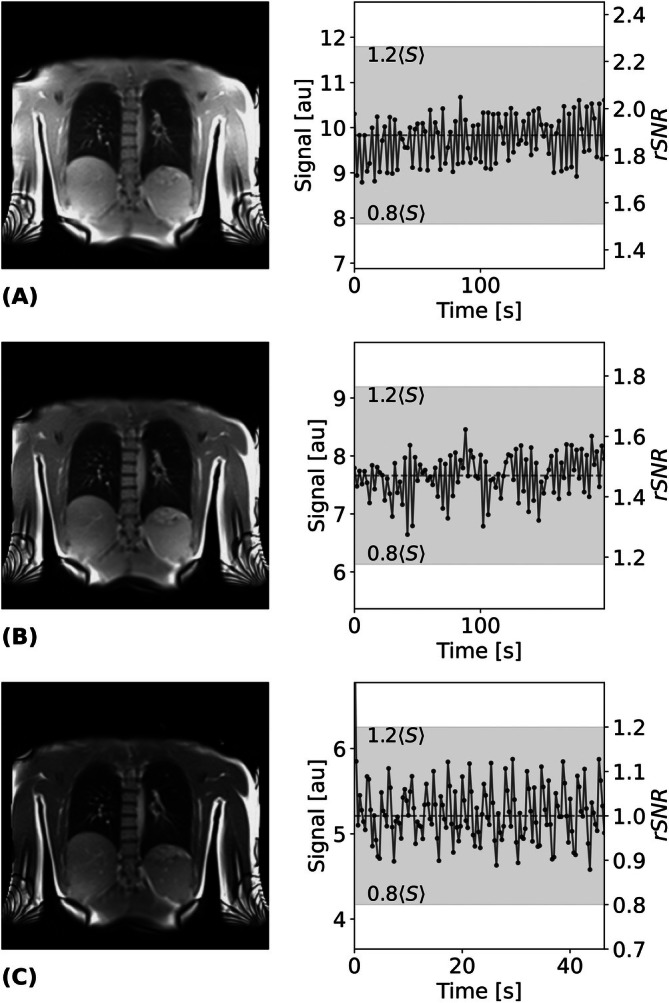
Example of CSC and MP MRI for one slice position of 10 mm slice thickness, acquired in free‐breathing. The images refer to the 100th image acquired in the timeline and the plots show the signal time course averaged over the segmented lung after image registration. The solid lines show the mean signal of all images used to calculate the rSNR for the CSC as compared to the time‐resolved scans. The same windowing was used for the images. (A) CSC with centric view ordering (100 images). (B) CSC with linear view ordering (100 images). (C) MP MRI with linear view ordering (140 images).

The time evolution of the average lung signal (and thus of the rSNR) for the CSC and the time‐resolved scans, acquired in free‐breathing and in a healthy volunteer, is illustrated for one coronal slice in Figure 3. Visually, it is evident that the CSC scan leads to a significant increase in signal and thus in rSNR as compared to the time‐resolved acquisition not only for the tissue outside the lung but also for the parenchymal tissue itself. Overall, the strongest increase in the rSNR is observed for the centric CSC scan (1.87±0.10), followed by the linear CSC (1.47±0.07) and the MP acquisition (1.00±0.07), as expected (see Figure 2). Over all volunteers, CSC as compared to the time‐resolved MP acquisition, leads to an average increase in the pulmonary rSNR by factors 1.47±0.16 and 2.04±0.21 for the linear and centric view ordering, respectively. Moreover, Figure 3 shows that the relative signal modulation over time is highest for the time‐resolved scan, whereas the signal variations are lower but similar for the two CSC scans. This observation likely reflects the fact that the time‐resolved signal is modulated by both respiratory and cardiac motion, whereas the CSC signal is, by design, intended to primarily reflect changes induced by the respiratory motion.

**FIGURE 4:**
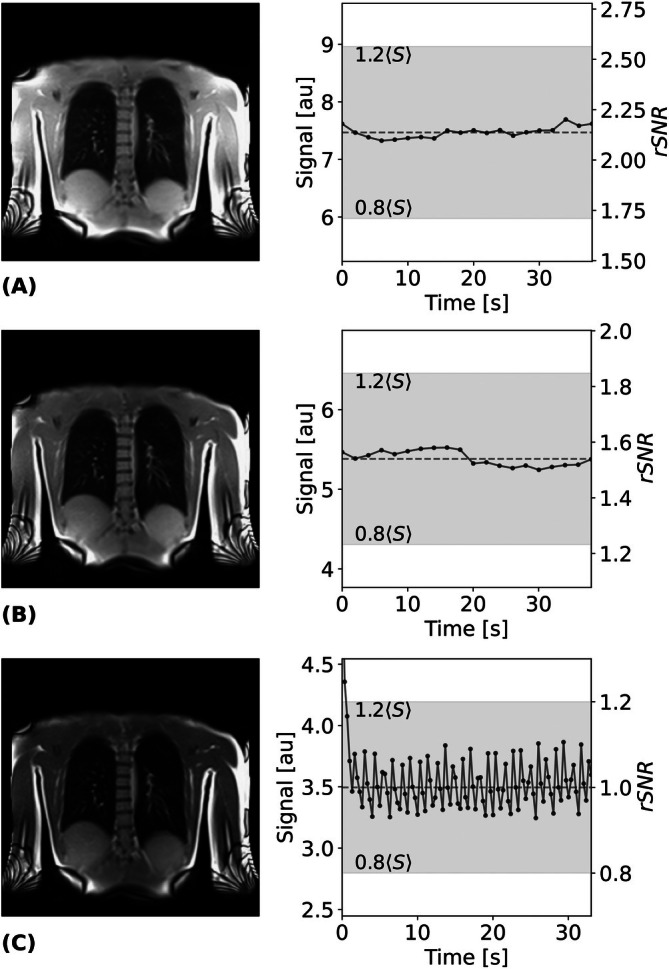
Example of CSC and MP MRI for one slice position of 10 mm slice thickness, acquired in breath hold. The images refer to the 20th image acquired in the timeline and the plots show the signal time course averaged over the segmented lung after image registration. The solid lines show the mean signal of all images used to calculate the rSNR for the CSC as compared to the time‐resolved scan. The same windowing was used for the images. (A) CSC with centric view ordering (one image/2 s; 20 images in total). (B) CSC with linear view ordering (one image/2 s; 20 images in total). (C) MP MRI with linear view ordering (three images/s; 100 images in total).

Figure 4 shows the same experiment for the same volunteer and slice position as in Figure 3, but the scans are now performed in breath‐hold. For the time‐resolved acquisition, irrespective of the breath‐holding, rapid signal modulations persist due to perfusion‐related signal, whereas for the CSC acquisitions, the fluctuations are nearly zero. Based on the coefficient of variation of the pulmonary signal, the time‐resolved sequence fluctuates about 5.7 times more than the centric CSC and about 4.3 times more than the linear CSC acquisition. Note that since the lung volume is increased during the inspiratory breath‐hold (Figure 4), the average lung signal is lower as compared to the tidal breathing scan (Figure 3).

**FIGURE 5:**
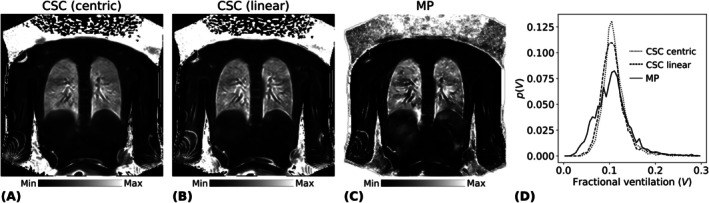
Ventilation image of a healthy volunteer using the coefficient of variation (CV) derived from the proposed CSC centric (A) and the CSC linear (B) sequence. For comparison, the fractional ventilation image from the MP analysis of a standard 2D time‐resolved bSSFP is shown in (C). Corresponding ventilation histograms are shown in (D). Note that the CV histograms were normalized to the mean of the fractional ventilation of the MP analysis for comparison purposes. All acquisitions were performed with a slice thickness of 10 mm.

Exemplary coronal ventilation images from a healthy lung are shown in Figure 5 for both CSC sampling schemes (linear and centric) using the CV analysis, as well as for the standard functional lung MRI using the MP analysis. Overall, there is a good visual agreement between the ventilation images produced by the CV for CSC and the MP results, although the results from the time‐resolved scan are less homogeneous, as reflected by the increased standard deviation of the fractional ventilation histograms: 0.044 for MP, 0.030 for CSC linear, and 0.025 for CSC centric. For both methods, however, non‐ventilated tissue outside of the lung is markedly suppressed.

In the following, only the centric CSC acquisition is further evaluated in patients, since it offers much higher signal as compared to the linear acquisition (see Figure 3) and images were free of artifacts in all volunteers. Overall, and similar to the volunteers, CSC offered for the patients a (2.17±0.32)—fold average increase in rSNR as compared to the time‐resolved scan. For three selected cases, corresponding ventilation images from the CSC and the time‐resolved scan are analyzed in some more detail below. Although the slice locations do not align completely, both approaches demonstrate excellent agreement in regional ventilation assessment.

**FIGURE 6:**
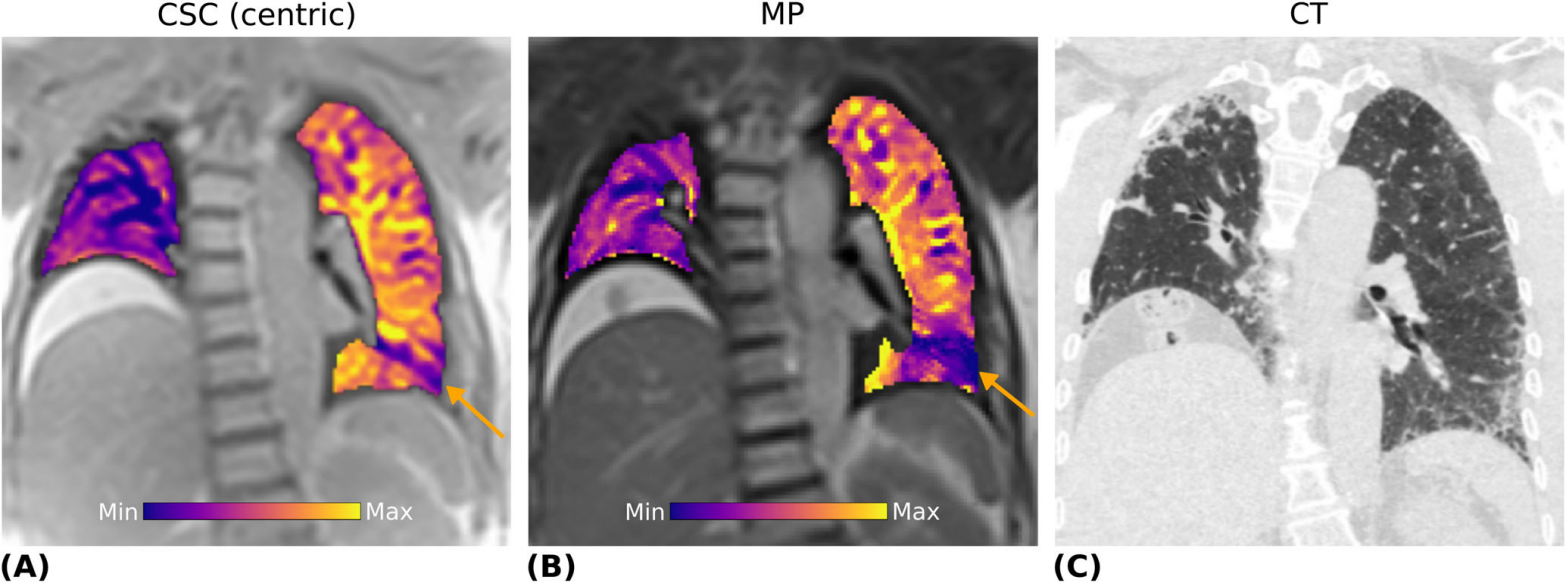
75‐year‐old female patient diagnosed with fibrosing hypersensitivity pneumonitis and elevated right hemidiaphragm. Ventilation maps indicate a strong functional impairment in the right lung, as well as hypoventilated regions affected by fibrosis in the lower lobe of the left lung (orange arrow). The ventilation maps were derived from the MP sequence (A) and the proposed CSC centric acquisition (B) with 10 mm slice thickness. The corresponding CT image demonstrates subpleural fibrosis in the left lower lobe (C).

Figure 6 shows the ventilation assessment of a patient diagnosed with fibrosing hypersensitivity pneumonitis and elevated right hemidiaphragm. The right lung exhibits limited ventilation, which is compensated by relative hyperventilation in the left lung. This distinct pattern is clearly identified by both MP and CSC analyses (Figure 6A,B). The corresponding CT image demonstrates subpleural fibrosis in the left lower and right upper lobes (Figure 6).

**FIGURE 7:**
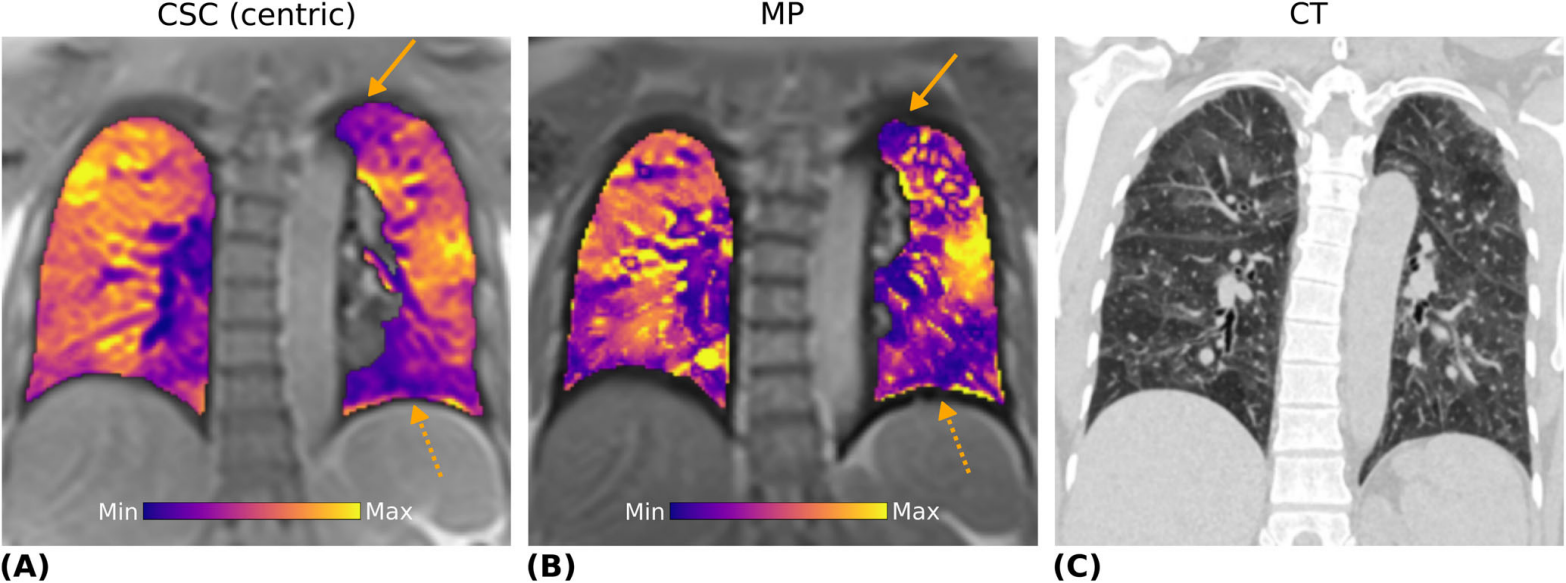
52‐year‐old female patient with T2‐low asthma. The CSC (A) and MP (B) ventilation maps of 10 mm slice thickness indicate peripheral functional impairment in the lower lobe of the left lung (dashed arrow) as well as in the apical region of the left lung (solid arrow). The corresponding CT image shows only mild inhomogeneities of lung parenchyma (C).

**FIGURE 8:**
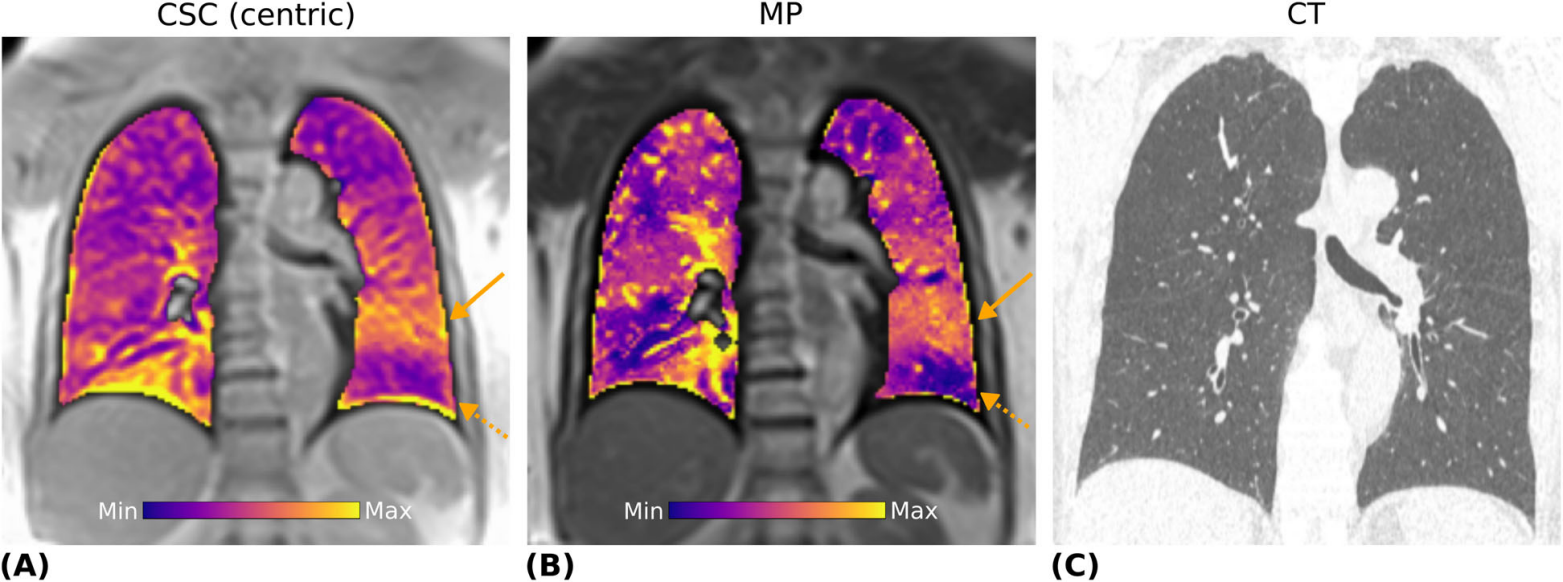
Fractional Ventilation of a 66‐year‐old female patient who underwent workup for a pulmonary nodule (not shown). Hypoventilated regions (dashed arrow) and hyperventilated regions (solid arrow) align in the CSC and MP analysis of 10 mm slice thickness. The ventilation maps were derived from the MP sequence (A) and the proposed CSC centric acquisition (B). The CT image shows normally appearing lung parenchyma (C).

The ventilation maps of a female patient diagnosed with T2‐low asthma is shown in Figure 7. Ventilation impairment in the lower lobe and in the apex of the left lung are detected in both the CSC and MP acquisition. The patient in Figure 8 underwent workup for a pulmonary nodule. Both the CSC and MP acquisitions detect hypo‐ and hyperventilated regions in the left lung with a strong spatial correspondence between both modalities.

**FIGURE 9:**
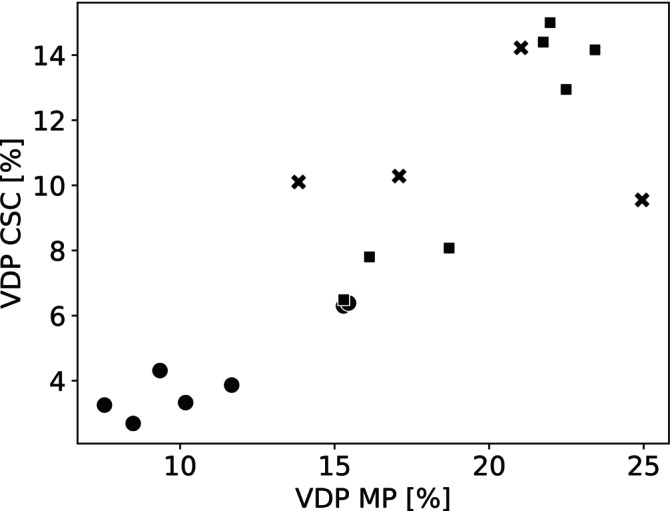
Comparison of ventilation defect percentages (VDP) between the time‐resolved sequence, using a MP analysis, and the CSC method using the CV. A correlation analysis yields a correlation of 0.88 over all volunteers and patients (data from volunteers is shown with circles, patient data with 10 mm slice thickness using cross‐marks, and patient scans with 12 mm slice thickness using squares).

Finally, for all volunteers and patients, the mean ventilation defect percentage (VDP) predicted by CSC was compared to the VDP of the MP analysis with the results shown in Figure 9. Between both methods, a high correlation of 0.88 was found that increased to 0.96, if the patient data acquired with a slice thickness of 10 mm, and thus lower SNR, was discarded.

## 4 Discussion

A novel image acquisition scheme, termed CSC, was introduced for functional lung MRI that performs imaging at thermal equilibrium. As a result, CSC yields more than two‐fold higher lung tissue signals as compared to conventional functional lung methods that sample the same slice with short waiting times (to allow in‐flow of unsaturated blood) and high temporal frequencies near the steady state [9, 10, 12, 13]. Generally, the novel concept is very versatile. For instance, not only variable flip angles might be used to further shape and optimize its signal response for centric view‐orderings, but also different SSFP kernels can be used. At 0.55T, field inhomogeneities are markedly reduced providing excellent prospects for artifact‐free bSSFP imaging, as demonstrated in this work. At high clinical field strength, such as 3T and above, however, bSSFP‐based imaging of the lung becomes unfeasible [23]. Here, an incoherent sampling approach could be used for CSC. This is of special notice, since with increasing magnetic field, the $T_{2}^{*}$‐related signal decay of GRE becomes more and more accentuated, thus leading to a decrease in SNR which can be counteracted using CSC.

In this work, we aimed to explore the upper limit of signal enhancement with CSC as compared to the common sequential, high‐frame rate, image sampling approach at a fixed slice position with contemporary functional lung MRI methods. To this end, the CSC protocol acquired the slices not only in an interleaved manner but also used a slice spacing of 100% to prevent any potential signal interference from adjacent slices, for example, due to overlapping slice profiles or due to in‐plane motion as a result of the breathing motion in the anterior–posterior direction. Moreover, partially saturated blood might lead to enhanced inflow effects, possibly leading to an increased and unwanted sensitivity to perfusion‐related signal modulations. Figure 2, however, indicates that, at 0.55T, a sampling period of 2 s is likely long enough to mitigate these effects, in principle also allowing the acquisition of adjacent slices without noticeable bias. This will allow to null the waiting time and thus lead to a further enhancement of the sampling efficiency of CSC; although currently the sequence dead time (due the waiting time) is about 25% of the total scan time—which seems acceptable.

Our study has several limitations. First, although not investigated in this work, CSC imaging is likely to be performed below the Nyquist frequency for physiological breathing rates. This makes it impossible to use a time domain analysis for ventilation imaging, such as with MP [10], or a retrospective sorting of images to resolve the breathing phase, such as with PREFUL [12]. Fractional ventilation was thus estimated using a statistical approach. In principle, however, a simple short and very low flip angle non‐selective 1D projection navigator scan in the head‐to‐foot direction could be acquired during the CSC waiting time. This will allow recording the breathing motion in parallel to the CSC slice sampling for retrospective sorting of images according to the respiratory cycle.

Second, the most evident drawback of the proposed CSC method is that it does not allow a simultaneous acquisition of both ventilation and perfusion images: the gain in signal comes at the expense of a loss of perfusion sensitivity. Thus, if also perfusion images are required, CSC can be combined with conventional functional lung MRI methods using time‐resolved acquisitions. The time‐resolved scan, however, then only needs to capture perfusion‐related signal modulations which should be possible in much shorter acquisition times; for example, in combination with a simple high‐pass filtering. In summary, for ventilation and perfusion imaging, CSC and time‐resolved MRI might be used in a complementary and synergetic way.

Lastly, a comparison of VDP between CSC and MP MRI in volunteers and a small, but inhomogeneous, patient cohort revealed a high correlation. At 0.55T, conventional lung functional MRI using the same slice thickness for the time‐resolved acquisition as for CSC resulted in more inhomogeneous ventilation maps in volunteers (see Figure 9). This finding is corroborated by the excellent correlation between the VDP of CSC and MP, if the patient data with 10 mm slice thickness is omitted. Nevertheless, a clinical validation of the proposed CSC acquisition against standard functional lung measures is missing and needs to be performed.

Future work will thus focus not only on a further optimization of the methods, addressing the above mentioned methodological aspects, but also on a clinical evaluation of CSC for ventilation MRI; especially in the presence of the so‐called “minus‐pathologies”, which are characterized by a decrease in local tissue density, for example, in emphysematous tissue destruction or hyperinflation caused by air‐trapping.

In summary, CSC can offer a significant signal boost whenever the lung signal is limited. This occurs in “minus‐pathologies”, at low field due to a low signal‐to‐noise ratio, and at high field where rapid $T_{2}^{*}$‐related signal decay is combined with incoherent gradient echo sampling. This overall presents excellent prospects for ventilation imaging of the lung in the clinical setting with increased sensitivity from low to high magnetic fields.

## 5 Conclusion

A novel image acquisition concept for native, proton‐based ventilation MRI was introduced, termed continuous slice cycling, CSC. In contrast to contemporary functional lung MRI methods, a considerable gain in SNR of about a factor of two can be achieved. As a result, CSC might offer excellent prospects for functional MRI from low to high magnetic fields and where signal from the lung is limited, such as at low field, or in combination with lung diseases that are characterized by a decrease in local tissue density.

## Funding

This research was supported by the Swiss National Science Foundation grant number 320030_219186.

## Supporting information


**Figure S1:** Ratio of lung signal intensities of the last three acquired images compared to the intensity of the first image as a function of the time between the acquisition of images of the same slice. The data are acquired in breath hold with a total of 10 images per sampling period *τ*.

## Data Availability

The data that support the findings of this study are available on request from the corresponding author. The data are not publicly available due to privacy or ethical restrictions.
