# Supplementary material for: Ventilation Imaging of the Lung at 0.55T With Continuous Slice Cycling
Source: Magn Reson Med. 2026 May 14;96(3):1235–44. doi: 10.1002/mrm.70436 (PMC13327447; doi:10.1002/mrm.70436)
Supplement: Supplementary file 1 — Figure S1: Ratio of lung signal intensities of the last three acquired images compared to the intensity of the first image as a function of the time between the acquisition of images of the same slice. The data are acquired in breath hold with a total of 10 images per sampling period τ. [file MRM-96-1235-s001.pdf]

Supporting Information

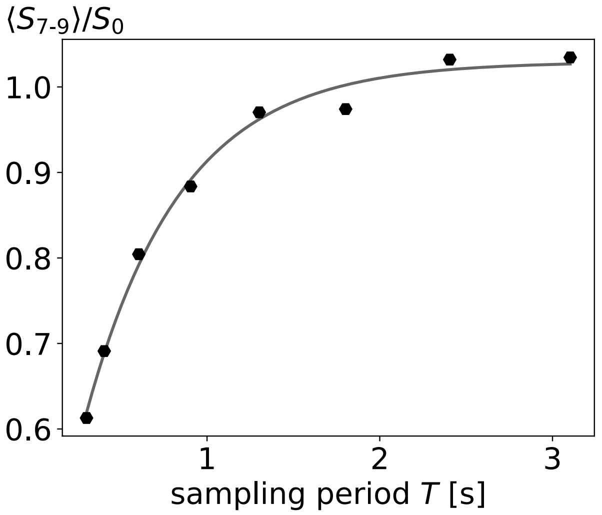

Figure S1: Ratio of lung signal intensities of the last three acquired images compared to the intensity of the first image as a function of the time between the acquisition of images of the same slice. The data is acquired in breath hold with a total of 10 images per sampling period τ.
